# Transition into daylight saving time influences the fragmentation of the rest-activity cycle

**DOI:** 10.1186/1740-3391-4-1

**Published:** 2006-01-19

**Authors:** Tuuli A Lahti, Sami Leppämäki, Sanna-Maria Ojanen, Jari Haukka, Annamari Tuulio-Henriksson, Jouko Lönnqvist, Timo Partonen

**Affiliations:** 1 Department of Mental Health and Alcohol Research, National Public Health Institute, Mannerheimintie 166, FI-00300 Helsinki, Finland; 2 Department of Psychiatry, Helsinki University Central Hospital, Helsinki, Finland; 3 Department of Psychiatry, University of Helsinki, Helsinki, Finland

## Abstract

**Background:**

Daylight saving time is widely adopted. Little is known about its influence on the daily rest-activity cycles. We decided to explore the effects of transition into daylight saving time on the circadian rhythm of activity.

**Methods:**

We monitored the rest-activity cycles with the use of wrist-worn accelerometer on a sample of ten healthy adults for ten days around the transition into summer time. Identical protocols were carried out on the same individuals in two consecutive years, yielding data on 200 person-days for analysis in this study.

**Results:**

There was no significant effect on the rest-activity cycle in the sample as a whole. Fragmentation of the rest-activity cycle was enhanced in a subgroup of persons having sleep for eight hours or less (P = 0.04) but reduced in those who preferred to sleep for more than eight hours per night (P = 0.05). The average level of motor activity was increased in persons having the morning preference for daily activity patterns (P = 0.01).

**Conclusion:**

Transition into daylight saving time may have a disruptive effect on the rest-activity cycle in those healthy adults who are short-sleepers or more of the evening type.

## Background

Daylight saving time (DST) is currently used in approximately 70 countries worldwide. The rationale for DST is to improve the match between the daylight hours and activity peaks of a population. Studies of traffic accidents have indicated that the increased availability of daylight hours in the evening under DST may either reduce [1,2] or increase [3] the number of motor vehicle crashes and pedestrian fatalities.

There are few reports of the impact of DST on the circadian rhythms or daily rest-activity cycles. In a study of 65 subjects [4], a disruptive effect was seen for five days after the termination of DST. Transitions into DST may be even more disruptive to the circadian time-keeping system. We hypothesize that they reduce the circadian amplitude and start driving the individual into a later activity phase. This may end with subsequent delays [5] on top of the delayed phase position and thereby compromise well-being.

## Methods

Ten healthy individuals, all free of psychotropic medication, participated in the study after giving a written informed consent. All subjects lived in Helsinki, Finland (60°12' N). None was a shift-worker nor crossed time zones during the study. Participants were asked to retain their normal or regular daily schedule during the study. Identical protocols were carried out twice on the same individuals, each using a personal and exclusive accelerometer throughout both study periods. We analyzed a total of 200 person-days for this study.

In 2003, DST was started on 30 March at 3 a.m. Rest-activity cycles were measured using wrist-worn accelerometers (Actiwatch-Plus^®^, Cambridge Neurotechnology Ltd., Cambridgeshire, UK) for a period of ten days from 24 March to 3 April 2003. In 2004, DST was started on 28 March at 3 a.m. Rest-activity cycles were measured using wrist-worn accelerometers for a period of ten days from 22 March to 1 April 2004. The participants wore the units all the time, except when bathing or swimming. The units were mounted on the non-dominant arm and positioned using a standardized protocol, recording the intensity, amount and duration of movement in all directions over 0.05 g, with the sampling epoch of 30 seconds. The sampling frequency of the units was 32 Hz at maximum, the filters being set to 3 to 11 Hz.

To assess the preference for daily activity patterns, the participants completed the Morningness-Eveningness Questionnaire [6]. This instrument includes 19 items estimating the preference for the timing of different activities and behaviors, whose sum yields the Morningness-Eveningness Score (MES), ranging from 16 to 86. The highest scores indicate definite morningness, the lowest definite eveningness. In addition, they were asked about the usual daily schedule and the estimate of how many hours of sleep they needed in order to feel refreshed. Each morning during the study period, participants wrote down the time of awakening that morning and the time of falling asleep the night before. Sleep debt was calculated as the difference between the preferred and actual length of sleep per night.

The participants were six women and four men, aged 32 to 70 years with the mean (standard deviation, SD) of 45.2 (10.7) years. They were assigned in groups by the preference for daily activity patterns (morning, intermediate or evening type), and the preferred length of sleep (more than 8 hours per night as long-sleepers, 8 hours or less per night as short-sleepers).

### Statistics

The data were extracted from the units and first analyzed with the software provided by the manufacturer (The Actiwatch Sleep Analysis 2001). Six variables were used for analysis: the highest 10 hours of activity, lowest 5 hours of activity, intra-daily stability, intra-daily variability, relative amplitude, and circadian period. Intra-daily stability (IS) quantifies the invariability between the days, i.e. the strength of coupling of the rhythm to supposedly stable environmental time-givers [7]. Intra-daily variability (IV) gives an indication of the fragmentation of the rhythm, i.e. the frequency and extent of transitions between rest and activity. The relative amplitude (RA) can be calculated from the most active 10-hour period (M10) and the least active 5-hour period (L5) in the average 24-hour pattern [8-10]. The circadian period (tau) was calculated with use of fast Fourier transform (FFT) analysis. FFT provides the frequency distribution of events, and the peaks on a FFT plot show correlations with the rhythmic events of activity. The peak correlation using the 1-minute resolution was analyzed as the estimate of the circadian period. The intra-daily stability, intra-daily variability, relative amplitude, and circadian period were calculated separately for the days before and those after the transition.

The significance of changes (before minus after) in these variables was analyzed using two-tailed, paired-samples t-test. These calculations were made using SPSS for Windows, Release 11.5.1 (SPSS Inc., Chicago, Illinois, USA). Partial correlation coefficients were calculated for the relevant variables (IS, IV, RA, tau), after controlling for age and sex. Differences between subgroups of the sample were analyzed using the analysis of variances with the subgroup as the independent factor.

The mesor (fitted mean), the acrophase (time of the peak of the fitted curve) and the amplitude (magnitude of the oscillation) were determined. To calculate these three variables, the raw data originally collected at 30-second intervals were merged into 30-minute intervals, including into analysis only those data points that comprised the 24-hour periods in full. Linear least-squares estimation was used for the data as follows.

Y (*t*) = *A*·(sin([2·π·*t*]/τ) + cos ([2·π·*t*]/τ)) + *M*,

where *A *= Amplitude, τ = period, *t *= time, and *M *= mesor.

The significance for changes in these variables was analyzed using the Welch two-sample t-test. These calculations were made using R, Version 1.8.1 .

Finally, to visualize the effect of a transition, the time series analysis was carried out using locally weighted regression, applying a decomposition procedure based on loess [11]. This method decomposes time series in three (trend, 24-hour, and the remainder) components using a sequence of smoothing operations, and is robust in detecting both trends and circadian variations.

## Results

Four participants had the preference for morning activities with the mean (standard deviation) MES of 61.0 (3.4), and six were of neither morning nor evening type with the mean (standard deviation) MES of 50.8 (3.8). Five reported the preferred length of sleep to be 8 hours or less, and five needed more than 8 hours of sleep per night. Table 1 presents the values at baseline and after transition for all participants, and Tables 2 and 3 for subgroups.

**Table 1:** Measures of the rest-activity cycle at baseline and after transition into DST

Variable	Before	After
	Mean (SD)	Mean (SD)

IS	0.67 (0.13)	0.75 (0.07)
IV	0.91 (0.26)	0.92 (0.23)
RA	0.93 (0.03)	0.92 (0.05)
tau (min)	1446.40 (9.00)	1440.60 (10.82)

Abbreviations: SD = standard deviation, IS = intra-daily stability, IV = intra-daily variability, RA = relative amplitude, tau = circadian period.

**Table 2:** Changes due to transition into DST among short- and long-sleepers

Variable	Mean	SD	P value
Short sleepers			
IS	-0.01	0.08	0.07
IV	-0.14	0.12	0.04
RA	+0.02	0.02	0.3
tau	+1.00	16.12	0.4
Long sleepers			
IS	-0.15	0.12	0.08
IV	+0.13	0.21	0.05
RA	-0.01	0.01	0.5
tau	+10.60	16.12	0.4

Abbreviations: SD = standard deviation, IS = intra-daily stability, IV = intra-daily variability, RA = relative amplitude, tau = circadian period.

**Table 3:** Changes due to transition into DST among morning and intermediate types

Variable	Mean	SD	P value
Morning types			
IS	-0.16	0.14	0.1
IV	+0.05	0.31	0.5
RA	+0.01	0.02	0.9
tau	+16.00	17.66	0.1
Intermediate types			
IS	-0.03	0.08	0.2
IV	-0.04	0.15	0.6
RA	+0.01	0.02	0.9
tau	-1.00	11.66	0.2

Abbreviations: SD = standard deviation, IS = intra-daily stability, IV = intra-daily variability, RA = relative amplitude, tau = circadian period.

In sample analysis, transition into DST caused no significant effect on the rest-activity cycle in the ten healthy adults (Table 1). Partial correlations indicated that the changes in the intra-daily variability had a negative association with those in the intra-daily stability (r = -0.78, P = 0.02). In addition, there was a negative and a positive correlation of the changes in the intra-daily variability with the changes in the relative amplitude and those in the circadian period respectively, but neither was a significant one. In the analysis of those data points that comprised only the 24-hour periods in full, the circadian amplitude and acrophase were similar after the transition into DST.

In subgroup analysis, the analyses of variance yielded no between-group difference in any of the variables under analysis, although the small size of the subgroups provided for limited statistical power. The intra-daily variability and relative amplitude were compromised after transition among short-sleepers but not among long-sleepers (Table 2). In morning types, the circadian period was shortened by 16 minutes on average and the intra-daily variability was reduced, these changes being opposite to those in evening types (Table 3). In addition, the average level of the rest-activity cycles was increased after transition among the morning types only (Welch two-sample t-test: t = 2.5, P = 0.01).

There was no marked difference in responses between men and women. However, the intra-daily variability was decreased in men but increased in women. The relative amplitude was increased and decreased respectively. These effects were opposite and might thus indicate a true sex-specific difference. Moreover, the intra-daily variability was decreased in older, whereas it was increased in younger individuals.

**Figure 1:**
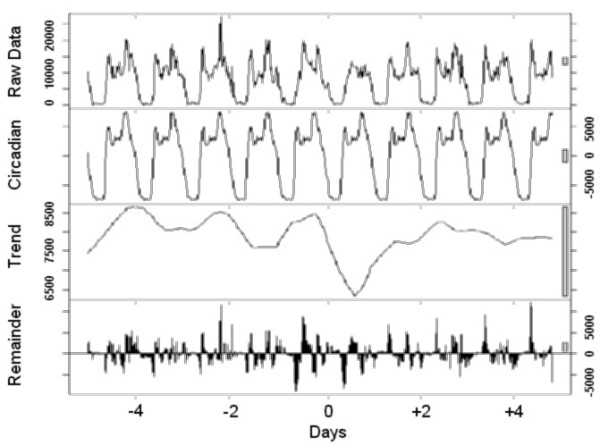
**All participants**. Data on the rest-activity cycle in 10 healthy persons for 10 days around the transition into DST in 2003. Graphics consist of four panels. The first panel from the top shows the original raw data, the second the circadian effect, the third the trend effect and the fourth the remainder. The remainder cannot be explained by the circadian nor trend effects. The scale bars to the right represent the corresponding unit in each figure.

**Figure 2:**
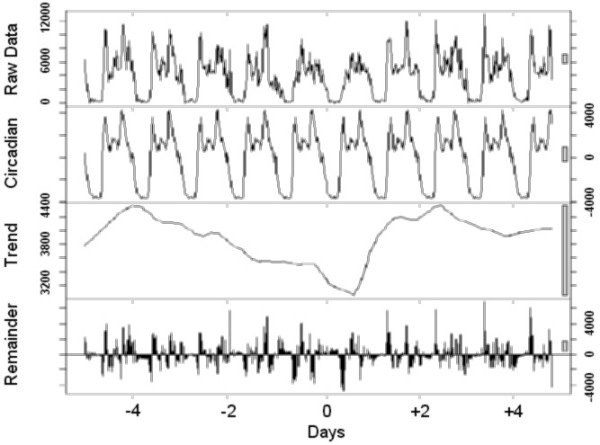
**Short-sleepers**. Data on the rest-activity cycle in short-sleepers for 10 days around the transition into DST in 2003.

**Figure 3:**
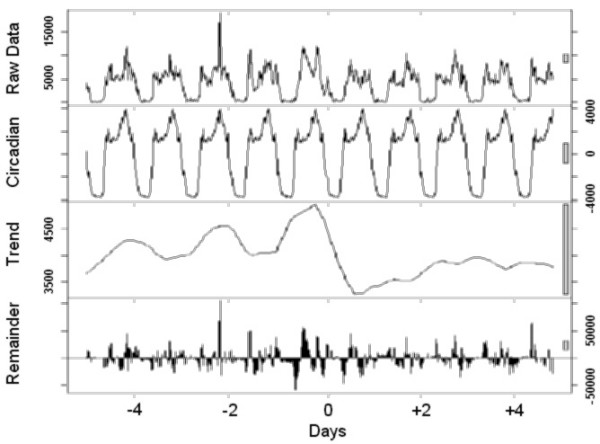
**Long-sleepers**. Data on the rest-activity cycle in long-sleepers for 10 days around the transition into DST in 2003.

**Figure 4:**
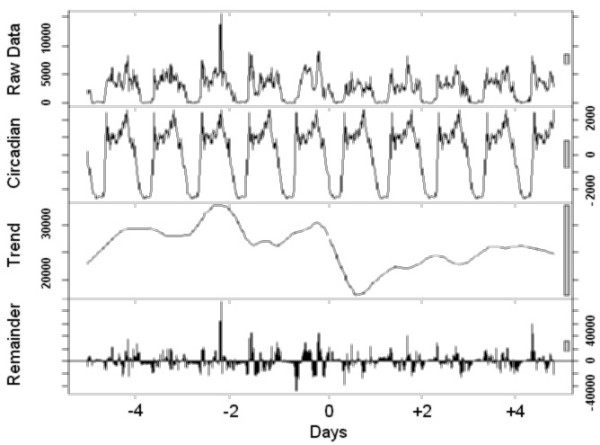
**Morning types**. Data on the rest-activity cycle in morning types for 10 days around the transition into DST in 2003.

**Figure 5:**
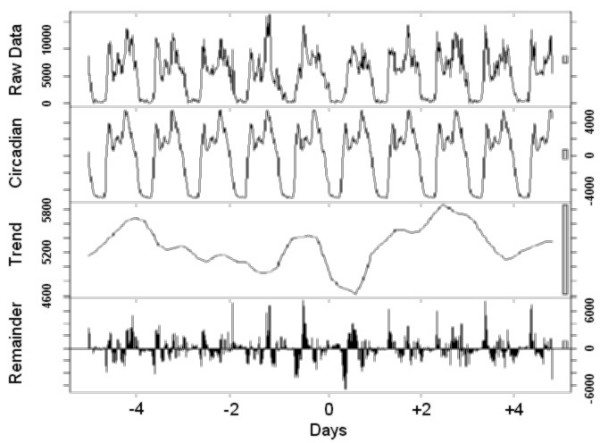
**Intermediate types**. Data on the rest-activity cycle in intermediate types for 10 days around the transition into DST in 2003.

The graphical presentations point out the rest-activity cycles and their changes in all individuals (Figure 1), those who preferred to sleep 8 hours or less (Figure 2) or more than 8 hours (Figure 3) per night, and those with the Morning (Figure 4) and Intermediate (Figure 5) preference for daily activity patterns. Figure 1 indicates that the activity peak during the day after the transition was smaller than the remaining peaks. Similar findings emerge from the subgroups.

## Discussion

There was no significant effect of transition into DST on the rest-activity cycle in our sample. We hypothesized that the transition would reduce the circadian amplitude and drive the individual into a later activity phase, but this did not happen. The cycle of rest-activity was obviously collapsed on the day after the transition from the remaining. This is in line with the reduction in the relative amplitude during four days after transition. However, there was no significant change in the circadian amplitude either. In addition, there was no evidence for a drive to a later phase position of motor activity, since after transition the circadian acrophase was not significantly different and the circadian period was shortened on average by only 5 min 48 sec.

Some interesting findings emerged from subgroup analysis. Here, our main finding was that transition into DST enhanced the fragmentation of the rest-activity cycle in persons who prefered to sleep for eight hours or less, but reduced it in those with more than eight hours of sleep per night. These significant changes in the intra-daily variability coincided with a decrease and an increase in the relative amplitude, respectively. Transition into DST appeared to jeopardize the circadian time-keeping system in short-sleepers by both increasing the intra-daily variability and reducing the relative amplitude of the rest-activity cycle, while the effects were rather positive on long-sleepers. Hence, our results indicate that long-sleepers gain from transitions into DST whereas short-sleepers tend to lose.

Our second finding concerns the morningness-eveningness typology. The intrinsic period of the circadian pacemaker is correlated not only with circadian phase, but also with wake-up and the behavioral trait of morningness-eveningness [12]. Individuals who have a preference for evening activities are likely to be affected more by a shortening of the external day, as they are predisposed to have sleep and mood disorders more frequently than the remaining [13]. In our study, there were no definite evening types. Subjects of intermediate type had changes in the rest-activity cycles that were not significant but anyway different from those of morning type. Transition into DST lengthened the circadian period and enhanced the fragmentation of the rest-activity cycle in the former, whereas the respective changes were opposite in the latter. Our finding that there was also an increase in the average level of motor activity in those having the morning preference, thereby gaining a benefit, agrees with the earlier literature. For them, it seems easier to shorten the circadian period in order to produce advances in the circadian phase and thereby terminate the day.

Third, our findings also point out that on the one hand women and on the other hand the younger are likely to react more easily to abrupt changes in the light-dark transitions or bedtime schedules. Their circadian time-keeping system may be less resilient and thereby make a difference in adaptation to and coping with distress.

A limitation of our study was the relatively small sample consisting of healthy subjects only in which great changes in the rest-activity cycles are not expected to occur. Yet, among some individuals, the fixed and abrupt advance in the external relative to internal time just by one hour did induce changes in the rest-activity cycles that remained for four days afterwards. These changes were modest and not significant, however, and their relevance or implications to treatment or counseling is not clear at the moment.

Our results cannot be generalized to the population at large nor to subjects with circadian rhythm related sleep or mood disorders. As DST affects everyone in a society, it is likely that on a population level many are affected more than the average in our study group. Transitions into DST may have no long-term effect on the circadian rhythms or rest-activity cycles in healthy individuals, but in patients the effect might be stronger.

For further exploration, we propose a trial analyzing the effects of transitions into DST and back to normal time in a clinical population, e.g. among the depressed who tend to have clear abnormalities in the circadian clockwork [14]. Earlier waking-up times in relation to sunrise appear to be associated with advances in the phase position of the circadian rhythms [15] and lower depression prevalence rates [16]. It is therefore likely that abrupt changes in the light-dark transitions such as those into DST will have more robust effects on affected than healthy subjects.

## Conclusion

Transition into DST may have a disruptive effect on the rest-activity cycle in those healthy adults who are short-sleepers or more of the evening type. This study needs replication on larger as well as clinical samples to analyze the effect among those with circadian rhythm related sleep or mood disorders.

## Competing interests

The author(s) declare that they have no competing interests.

## Authors' contributions

**TAL **made contributions to the analysis and interpretation of data and to the drafting and writing of the manuscript.

**SL **participated in the planning of the study, in the analysis of data, and in the drafting of the manuscript.

**S-MO **made contributions to statistical modeling and analysis and to the drafting of the manuscript.

**JH **made contributions to statistical modeling and analysis and to the drafting of the manuscript.

**AT-H **participated in the planning of the study and in the drafting of the manuscript.

**JL **participated in the planning of the study and in the drafting of the manuscript.

**TP **participated in the planning of the study, in the analysis of data, and in the drafting of the manuscript.

## References

[B1] Ferguson SA, Preusser DF, Lund AK, Zador PL, Ulmer RG (1995). Daylight saving time and motor vehicle crashes: the reduction in pedestrian and vehicle occupant fatalities. Am J Public Health.

[B2] Coate D, Markowitz S (2004). The effects of daylight and daylight saving time on US pedestrian fatalities and motor vehicle occupant fatalities. Accid Anal Prev.

[B3] Coren S (1996). Accidental death and the shift to daylight saving time. Percept Mot Skills.

[B4] Monk TH, Folkard S (1976). Adjusting to the changes to and from Daylight Saving Time. Nature.

[B5] Beersma DGM, Daan S, Hut RA (1999). Accuracy of circadian entrainment under fluctuating light conditions: contributions of phase and period responses. J Biol Rhythms.

[B6] Horne JA, Östberg O (1976). A self-assessment questionnaire to determine morningness-eveningness in human circadian rhythms. Int J Chronobiol.

[B7] Van Someren EJW, Swaab DF, Colenda CC, Cohen W, McCall WV, Rosenquist PB (1999). Bright light therapy: improved sensitivity to its effects on rest-activity rhythms in Alzheimer patients by application of nonparametric methods. Chronobiol Int.

[B8] Witting W, Kwa IH, Eikelenboom P, Mirmiran M, Swaab DF (1990). Alterations in the circadian rest-activity rhythm in aging and Alzheimer's disease. Biol Psychiatry.

[B9] Van Someren EJW, Lijzenga C, Mirmiran M, Swaab DF (1997). Long-term fitness training improves the circadian rest-activity rhythm in healthy elderly males. J Biol Rhythms.

[B10] Van Someren EJW, Scherder EJA, Swaab DF (1998). Transcutaneous electrical nerve stimulation (TENS) improves circadian rhythm disturbances in Alzheimer's disease. Alzheimer Dis Assoc Disord.

[B11] Cleveland RB, Cleveland WS, McRae JE, Terpenning I (1990). STL: a seasonal-trend decomposition procedure based on loess. J Official Stat.

[B12] Duffy JF, Rimmer DW, Czeisler CA (2001). Association of intrinsic circadian period with morningness-eveningness, usual wake time, and circadian phase. Behav Neurosci.

[B13] Taillard J, Philip P, Chastang JF, Diefenbach K, Bioulac B (2001). Is self-reported morbidity related to the circadian clock?. J Biol Rhythms.

[B14] Bunney WE, Bunney BG (2000). Molecular clock genes in man and lower animals: possible implications for circadian abnormalities in depression. Neuropsychopharmacology.

[B15] Burgess HJ, Eastman CI A late wake time phase delays the human dim light melatonin rhythm. Neurosci Lett.

[B16] Olders H (2003). Average sunrise time predicts depression prevalence. J Psychosom Res.

